# Taletrectinib in ROS1+ non–small cell lung cancer: a cost-effectiveness analysis in the United States

**DOI:** 10.3389/fphar.2026.1684603

**Published:** 2026-04-13

**Authors:** Gengwei Huo, Xiaoyan Wang, Ying Song, Yi Tian, Peng Chen

**Affiliations:** 1 Department of Thoracic Oncology, Tianjin Medical University Cancer Institute and Hospital, National Clinical Research Center for Cancer, Key Laboratory of Cancer Prevention and Therapy of Tianjin, Tianjin’s Clinical Research Center for Cancer, Tianjin, China; 2 Tianjin Medical University Cancer Institute and Hospital, National Clinical Research Center for Cancer, Key Laboratory of Cancer Prevention and Therapy of Tianjin, Tianjin’s Clinical Research Center for Cancer, Tianjin, China; 3 Department of Pharmacy, Jining NO.1 People’s Hospital, Jining, Shandong, China; 4 Medical Insurance Office, Tianjin Medical University Cancer Institute & Hospital, Tianjin, China

**Keywords:** cost-effectiveness, NSCLC, partitioned survival model, ROS1, taletrectinib

## Abstract

**Objective:**

To evaluate the cost-effectiveness by comparing four treatment strategies for ROS1-positive non-small cell lung cancer (NSCLC): first-line taletrectinib prior to chemotherapy, second-line taletrectinib following chemotherapy, second-line taletrectinib after crizotinib, and chemotherapy alone.

**Methods:**

A partitioned survival model was constructed to analyze the clinical outcomes and healthcare expenditures associated with the four treatment strategies. Costs and utility values were obtained from reputable literature sources and publicly available cost databases. Cost-effectiveness was evaluated under different taletrectinib cost scenarios to determine the impact of taletrectinib pricing on the four treatment strategies.

**Results:**

All three taletrectinib-containing treatment strategies exceeded the established U.S. willingness-to-pay (WTP) threshold of $150,000 per QALY, indicating they were not cost-effective. Among the evaluated strategies, first-line taletrectinib administered prior to chemotherapy provided the largest gain in quality-adjusted life years (QALYs), adding 4.61372 QALYs. However, this came at a substantial additional cost of $1,263,183.95, resulting in an incremental cost-effectiveness ratio (ICER) of $273,789 per QALY compared to chemotherapy alone. The second-line use of taletrectinib following crizotinib provided a more favorable cost-effectiveness profile. It yielded an additional 2.03260 QALYs at an additional cost of $441,371.10, leading to an ICER of $217,146 per QALY compared to chemotherapy. While all three strategies demonstrated ICERs above the WTP threshold, the second-line approach following crizotinib offered the most favorable, though still not cost-effective, cost-effectiveness compared to the other options. Conversely, second-line taletrectinib administered following chemotherapy exhibited the poorest cost-effectiveness, with an ICER of $587,663 per QALY. Furthermore, the sensitivity analysis highlighted that the cost of taletrectinib was the primary driver of the ICER’s unfavorable results.

**Conclusion:**

Based on current pricing, none of the taletrectinib-containing treatment strategies were found to be cost-effective for patients with ROS1-positive NSCLC compared to chemotherapy alone, as their ICERs exceeded the established WTP threshold. Nevertheless, considering the individual patient’s priorities, a personalized approach to treatment decisions can be adopted. For patients prioritizing the maximization of QALYs and with the necessary financial resources, first-line taletrectinib prior to chemotherapy may be a preferred option. Conversely, for those with limited financial capacity or in contexts prioritizing cost-containment, second-line taletrectinib after crizotinib could be more suitable.

## Introduction

In 2026, projections estimate that the United States will see approximately 229,410 new cases of lung cancer, with an estimated 124,990 deaths directly attributable to this malignancy (Siegel et al., 2026). Non-small cell lung cancer (NSCLC) accounts for the vast majority of these cases, representing approximately 85% of all lung cancer diagnoses (de Groot et al., 2018). Unfortunately, NSCLC is often associated with a poor prognosis, primarily due to the high proportion of patients diagnosed at an advanced stage or with evidence of metastasis (Reade and Ganti, 2009).

*ROS1* proto-oncogene tyrosine-protein kinase 1 (*ROS1*) gene fusions are identified in approximately 1%–2% of NSCLC cases (Lipson et al., 2012; Tsuta et al., 2014; Drilon et al., 2018). These fusions are more frequently detected in adenocarcinoma, advanced-stage disease, and among younger patients with a history of minimal or no smoking (Lin and Shaw, 2017). The resulting constitutive activation of ROS1 kinase activity triggers the upregulation of MAPK/ERK, PI3K/AKT, and JAK/STAT signaling pathways, promoting cellular proliferation, survival, and migration (Roskoski Jr, 2017; Rosell et al., 2023).

Taletrectinib is an oral, selective, central nervous system active, next-generation ROS1 tyrosine kinase inhibitor (TKI). On 11 June 2025, the Food and Drug Administration (FDA) approved taletrectinib for adult patients with locally advanced or metastatic ROS1-positive NSCLC, based on an integrated analysis of the phase II, regional TRUST-I and global TRUST-II trials (FDA, 2025). The trial results demonstrated durable responses, prolonged PFS, and favorable safety in both TKI-naïve and pretreated patients. For TKI-naïve patients, the median progression-free survival (PFS) was 45.6 months, and the overall response rate (ORR) was 88.8%. For TKI-pretreated patients, the median PFS was 9.7 months, and the ORR was 55.8% (Pérol et al., 2025).

Despite demonstrating durable clinical activity, taletrectinib often results in patients requiring prolonged treatment courses. This extended treatment, however, comes at a substantial financial burden, with a monthly cost of approximately $28,811.7 (Drugs.com, 2025). Healthcare providers and insurance companies therefore face the critical task of making informed decisions about prescribing and coverage. To support these decisions, a thorough understanding of the drug’s pharmacoeconomic characteristics is essential. This involves evaluating its cost-effectiveness—specifically, how its costs compare to its benefits in terms of improved health outcomes and quality of life. Such an assessment is particularly crucial within the context of the high healthcare costs prevalent in the U.S. Ultimately, a comprehensive pharmacoeconomic evaluation of taletrectinib will help clarify whether the benefits of this innovative treatment justify its high price, thereby enabling evidence-based decision-making for clinicians and payers alike.

Based on the National Comprehensive Cancer Network (NCCN) guidelines, taletrectinib can be used as either a first-line or second-line treatment option for ROS1-positive NSCLC (NCCN, 2025). In this study, a cost-effectiveness analysis was conducted from the perspective of the U.S. healthcare payer to evaluate the economic value of taletrectinib. Four treatment strategies were compared: first-line taletrectinib prior to chemotherapy, second-line taletrectinib following chemotherapy, second-line taletrectinib after crizotinib, and chemotherapy alone. The analysis estimated total costs, quality-adjusted life-years (QALYs), and incremental cost-effectiveness ratios (ICERs), providing economic evidence to inform clinical practice and healthcare decision-making.

## Materials and methods

### Model structure

A partitioned survival model was constructed using TreeAge Pro 2022 software (TreeAge Software, Massachusetts, United States). The model had a cycle length of 3 weeks. The analysis estimated QALYs, total costs, and ICERs. A lifetime horizon was applied, beginning at a median age of 56 years and extending to 78 years, consistent with the median age reported in the TRUST trial and the average life expectancy in the United States (Arias et al., 2025).

### Treatment strategies

The treatment strategies included in the model were based on clinical data from the TRUST trial and recommendations from the NCCN guidelines (NCCN, 2025; Pérol et al., 2025).

Four treatment strategies were evaluated.

1. First-line taletrectinib prior to chemotherapy:

Patients received taletrectinib as first-line therapy. Upon disease progression, treatment switched to cisplatin–pemetrexed doublet chemotherapy, followed by docetaxel chemotherapy.

2. Second-line taletrectinib following chemotherapy:

Patients initially received first-line cisplatin–pemetrexed doublet chemotherapy. After disease progression, taletrectinib was administered as second-line therapy, followed by docetaxel chemotherapy.

3. Second-line taletrectinib after crizotinib:

Patients received crizotinib as first-line therapy. After disease progression, taletrectinib was administered as second-line therapy, followed by cisplatin–pemetrexed doublet chemotherapy and docetaxel chemotherapy.

4. Chemotherapy alone:

Patients received cisplatin–pemetrexed doublet chemotherapy followed by docetaxel chemotherapy without targeted therapy.

**FIGURE 1:**
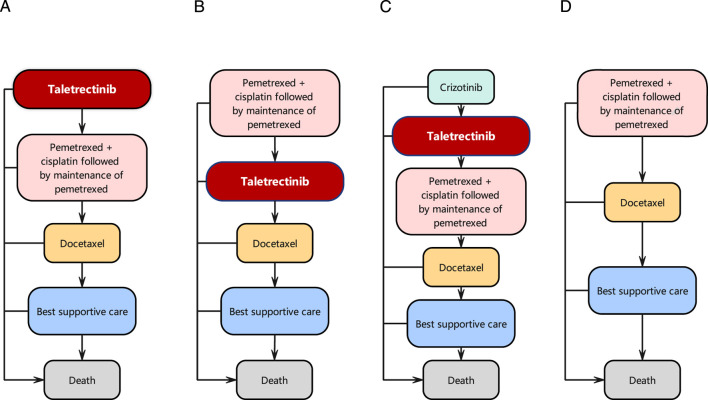
The treatment sequences utilized in the model are as follows: **(A)** First-line taletrectinib prior to chemotherapy; **(B)** Second-line taletrectinib following chemotherapy; **(C)** Second-line taletrectinib after crizotinib; **(D)** Chemotherapy only.

Patients who experienced disease progression after docetaxel chemotherapy were assumed to have reached an advanced stage of the disease and subsequently received best supportive care (BSC) until death (Figure 1).

### Drug dosing

Drug dosing schedules were obtained from the TRUST, PROFILE 1001, IFCT-1103 ULTIMATE, and PARAMOUNT trials. Taletrectinib was administered orally at 600 mg once daily until disease progression (Pérol et al., 2025). Crizotinib was administered orally at 250 mg twice daily (Shaw et al., 2019). Pemetrexed was administered at a dose of 500 mg/m^2^ until disease progression and was given concurrently with cisplatin (75 mg/m^2^) on day 1 from cycle 1 to cycle 4 (Paz-Ares et al., 2012). Docetaxel was administered at 75 mg/m^2^ every 3 weeks for four cycles (Cortot et al., 2020). For drugs dosed based on body surface area, costs were calculated assuming a standard body surface area (BSA) of 1.82 m^2^ (Lin et al., 2020).

### Survival risk calculation

Kaplan–Meier (K–M) curves for progression-free survival (PFS) were extracted from the TRUST, PROFILE 1001, IFCT-1103 ULTIMATE, and PARAMOUNT trials. The overall survival (OS) curve for best supportive care (BSC) was obtained from a previously published study (Shepherd et al., 2000; Paz-Ares et al., 2012; Shaw et al., 2019; Cortot et al., 2020; Pérol et al., 2025). PFS data for both the first-line taletrectinib prior to chemotherapy strategy and the second-line taletrectinib following chemotherapy strategy were derived from the ROS1 TKI-naïve cohort of the TRUST trial. In contrast, PFS data for the second-line taletrectinib after crizotinib strategy were obtained from the ROS1 TKI-pretreated cohort of the same trial. The K–M curves were digitized using GetData Graph Digitizer. Individual patient data were reconstructed using the algorithm proposed by Hoyle and Heneley (2011), Wan et al. (2015). Parametric survival models—including Weibull, exponential, Gompertz, log-normal, log-logistic, gamma, and generalized gamma distributions—were fitted to the reconstructed datasets. The optimal models were selected based on the Akaike information criterion (AIC) and Bayesian information criterion (BIC) (Supplementary Table S1; Supplementary Figure S1). The parameters of the selected survival models were subsequently used to estimate transition probabilities between health states in the model. In addition, age-specific background mortality rates derived from U.S. life tables were incorporated as lower-bound constraints for survival probabilities (Supplementary Table S2) (Arias et al., 2025).

### Health utility

Treatment effectiveness was measured in quality-adjusted life-years (QALYs), which were calculated by assigning utility values ranging from 0 (death) to 1 (perfect health) to different health states (Whitehead and Ali, 2010). The model included the following health states: progression-free disease, disease progression after first-line treatment, disease progression after second-line treatment, disease progression after third-line treatment, and death. Utility values for these health states were 0.71, 0.67, 0.59, 0.46, and 0, respectively (Chouaid et al., 2013). The impact of adverse events (AEs) on QALYs was incorporated by applying disutility values weighted by the incidence rates of AEs (Xu et al., 2022; Cao et al., 2024; Wu et al., 2025). The aggregated disutility was applied during the first treatment cycle to reflect the short-term impact of treatment-related AEs (Su et al., 2021) (Table 1).

**TABLE 1:** Model parameters and distributions.

Variable	Baseline value (References)	Range		Distribution
		Minimum	Maximum	
Exponential PFS survival model with taletrectinib 1st-line	rate = 0.0205875	—	—	—
Log-normal PFS survival model with taletrectinib 2nd-line	meanlog = 2.23795 sdlog = 1.19762	—	—	—
Log-normal PFS survival model with crizotinib	meanlog = 3.06187 sdlog = 1.46181	—	—	—
Gen. gamma PFS survival model with pemetrexed	mu = 1.824618 sigma = 0.500332 Q = −0.853057	—	—	—
Gamma PFS survival model with docetaxel	shape = 1.597317 rate = 0.350431	—	—	—
Log-logistic OS survival model with BSC	shape = 2.25761 scale = 4.73069	—	—	—
Grade ≥3 AEs incidence in taletrectinib				
Decreased neutrophil count	0.045 (Pérol et al., 2025)	0.036	0.054	Beta
Increased AST	0.063 (Pérol et al., 2025)	0.0504	0.0756	Beta
Increased ALT	0.088 (Pérol et al., 2025)	0.0704	0.1056	Beta
Grade ≥3 AEs incidence in crizotinib	​	​	​	​
Vomiting	0.04 (Shaw et al., 2019)	0.032	0.048	Beta
Elevated transaminases	0.04 (Shaw et al., 2019)	0.032	0.048	Beta
Neutropenia	0.09 (Shaw et al., 2019)	0.072	0.108	Beta
Grade ≥3 AEs incidence in pemetrexed + cisplatin chemotherapy				
Anemia	0.04 (Paz-Ares et al., 2012)	0.032	0.048	Beta
Decreased neutrophil count	0.04 (Paz-Ares et al., 2012)	0.032	0.048	Beta
Fatigue	0.04 (Paz-Ares et al., 2012)	0.032	0.048	Beta
Grade ≥3 AEs incidence in docetaxel chemotherapy				
Decreased neutrophil count	0.455 (Cortot et al., 2020)	0.364	0.546	Beta
Febrile neutropenia	0.073 (Cortot et al., 2020)	0.0584	0.0876	Beta
Anemia	0.073 (Cortot et al., 2020)	0.0584	0.0876	Beta
Asthenia	0.055 (Cortot et al., 2020)	0.044	0.066	Beta
Utility				
Progression-free disease	0.71 (Chouaid et al., 2013)	0.568	0.852	Beta
Progressed disease after first-line	0.67 (Chouaid et al., 2013)	0.536	0.804	Beta
Progressed disease after second-line	0.59 (Chouaid et al., 2013)	0.472	0.708	Beta
Progressed disease after third-line	0.46 (Chouaid et al., 2013)	0.368	0.552	Beta
AEs disutility				
Elevated transaminases/increased AST/ALT	0.05 (Wu et al., 2025)	0.040	0.060	Beta
Anemia	0.32 (Cao et al., 2024)	0.256	0.384	Beta
Decreased neutrophil count	0.46 (Cao et al., 2024)	0.368	0.552	Beta
Febrile neutropenia	0.50 (Cao et al., 2024)	0.400	0.600	Beta
Fatigue	0.20 (Cao et al., 2024)	0.160	0.240	Beta
Asthenia	0.20 (Cao et al., 2024)	0.160	0.240	Beta
Vomiting	0.21 (Xu et al., 2022)	0.168	0.252	Beta
Drug cost (U.S. $)				
Taletrectinib/200 mg	320.13 (Drugs.com, 2025)	256.10	384.16	Gamma
Crizotinib/250 mg	387.16 (Drugs.com, 2025)	309.73	464.59	Gamma
Pemetrexed/500 mg	51.38 (Drugs.com, 2025)	41.10	61.66	Gamma
Cisplatin/50 mg	10.55 (Drugs.com, 2025)	8.44	12.66	Gamma
Docetaxel/160 mg	137.77 (Drugs.com, 2025)	110.22	165.32	Gamma
AEs cost (U.S. $)				
Elevated transaminases/increased AST/ALT	9,132.00 (Mudumba et al., 2025)	7,305.60	10,958.40	Gamma
Anemia	25,269.00 (Mudumba et al., 2025)	20,215.20	30,322.80	Gamma
Decreased neutrophil count/febrile neutropenia	21,429.00 (Mudumba et al., 2025)	17,143.20	25,714.80	Gamma
Fatigue	48.53 (Cao et al., 2024)	38.82	58.24	Gamma
Asthenia	48.53 (Cao et al., 2024)	38.82	58.24	Gamma
Vomiting	787.72 (Xu et al., 2022)	630.18	945.26	Gamma
Cost of administration (U.S. $)	106.33 (Cao et al., 2024)	85.06	127.60	Gamma
Cost of CT imaging (U.S. $)	541.00 (Wu et al., 2025)	432.80	649.20	Gamma
Cost of brain MRI imaging (U.S. $)	337.00 (Wu et al., 2025)	269.60	404.40	Gamma
Cost of laboratory testing (U.S. $)	23.75 (Wu et al., 2025)	19.00	28.50	Gamma
Cost of end-of-life care (U.S. $)	11,573.94 (Wu et al., 2025)	9,259.15	13,888.73	Gamma
Cost of physician visit (U.S. $)	133.61 (Cao et al., 2024)	106.89	160.33	Gamma
BSC per cycle (U.S. $)	781.58 (Wu et al., 2025)	625.26	937.90	Gamma
Patients’ body surface area (m^2^)	1.82 (Lin et al., 2020)	1.456	2.184	Normal
Discount rate (%)	3 (Lin et al., 2020)	0	5	Triangular

AEs, adverse effects; BSC, best supportive care; OS, overall survival; PFS, progression-free survival.

### Cost estimates

Cost estimation followed the framework described by Tumeh et al. (2005). Costs included drug acquisition, administration, treatment of adverse events, end-of-life palliative care, and best supportive care (BSC). Cost inputs were obtained from previously published studies and publicly available drug pricing sources (Table 1) (Xu et al., 2022; Cao et al., 2024; Drugs.com, 2025; Mudumba et al., 2025; Wu et al., 2025). Only Grade 3 or higher adverse events with an incidence rate greater than 4% were included in the analysis (Huo et al., 2024). Consistent with common practice in cost-effectiveness analyses, AE management costs were assumed to occur only during the first cycle following their onset and were treated as one-time costs (Su et al., 2021). Disease monitoring included contrast-enhanced computed tomography (CT) and brain magnetic resonance imaging (MRI). CT scans were conducted every two cycles during the first eight treatment cycles, every three cycles from cycles 9 to 26, and every four cycles thereafter. For the 33.7% of patients with baseline brain metastases, brain imaging continued throughout treatment using the same schedule as systemic tumor assessments (Pérol et al., 2025). Laboratory testing and physician visit costs were included when intravenous chemotherapy or contrast-enhanced CT imaging was performed (Ding et al., 2021). All cost inputs were adjusted for inflation using the U.S. Consumer Price Index (CPI) and were reported in 2025 U.S. dollars (Halfhill, 2025).

### Sensitivity analyses

One-way sensitivity analyses were conducted by varying each model parameter by ±20% from its baseline value. The resulting variations in model outcomes were illustrated using tornado diagrams. Probabilistic sensitivity analysis (PSA) was performed using 1,000 Monte Carlo simulations, in which all parameters were simultaneously varied according to their assigned probability distributions. Cost parameters were modeled using gamma distributions, whereas proportions and utility parameters followed beta distributions (Table 1). The formulas used to derive the parameters for each distribution are provided in Supplementary Table S4. Cost-effectiveness acceptability curves (CEACs) and scatter plots were generated to evaluate the probability that each treatment strategy would be cost-effective at different willingness-to-pay thresholds.

### Statistical analysis

A half-cycle correction was applied in the model, and both costs and life-years were discounted at an annual rate of 3% (Lin et al., 2020). All analyses were conducted using R software (version 4.2.1). The model estimated costs, utility values, QALYs, and ICERs. A willingness-to-pay (WTP) threshold of $150,000 per QALY was adopted to determine cost-effectiveness (Rabin and de Charro, 2001; Neumann et al., 2014; Lin et al., 2020). ICERs were calculated to compare the four treatment strategies and represent the ratio of differences in costs to differences in effectiveness. The ICER was calculated as:

$$ICER=\frac{C1-C2}{E1-E2}$$

where *C* represents the cost associated with each treatment strategy and *E* represents its effectiveness.

## Results

### Model validation

PFS and OS outcomes were simulated based on data extracted from the K-M survival curves of each clinical trial. The results of these simulations demonstrated a high level of concordance with the published data, with no obvious differences identified (Supplementary Table S3). The alignment between the modeled survival distributions and the observed data from the K-M analyses is visually depicted in Supplementary Figure S1.

### Base case results

Among the evaluated taletrectinib-containing strategies, “First-line taletrectinib prior to chemotherapy” offered the greatest gains in QALYs, delivering an additional 4.61372 QALYs at an incremental cost of $1,263,183.95 compared to “chemotherapy alone”, resulting in an ICER of $273,789 per QALY.

“Second-line taletrectinib after crizotinib” demonstrated the lowest ICER among the taletrectinib-containing strategies. This approach provided an additional 2.03260 QALYs at an incremental cost of $441,371.10, leading to an ICER of $217,146 per QALY compared to “chemotherapy alone”.

Conversely, “second-line taletrectinib following chemotherapy” exhibited the poorest cost-effectiveness among the options. While yielding an additional 1.71032 QALYs, its substantial incremental cost of $1,005,090.93 resulted in the highest ICER of $587,663 per QALY compared to “chemotherapy alone”.

However, all three taletrectinib-containing treatment strategies consistently showed ICERs exceeding the established U.S. WTP threshold of $150,000 per QALY (Table 2).

**TABLE 2:** Base-case results of the model.

Arm	Costs (U.S. $)	△Costs (U.S. $)	QALYs	△QALYs	ICER (U.S. $/QALY)
Chemotherapy alone	54,996.28	-	1.32080	-	-
First-line taletrectinib prior to chemotherapy	1,318,180.23	1,263,183.95	5.93452	4.61372	273,789
Second-line taletrectinib following chemotherapy	1,060,087.21	1,005,090.93	3.03112	1.71032	587,663
Second-line taletrectinib after crizotinib	496,367.39	441,371.10	3.35340	2.03260	217,146

ICER, incremental cost-effectiveness ratio; QALY, quality-adjusted life-years.

### Sensitivity analyses

**FIGURE 2:**
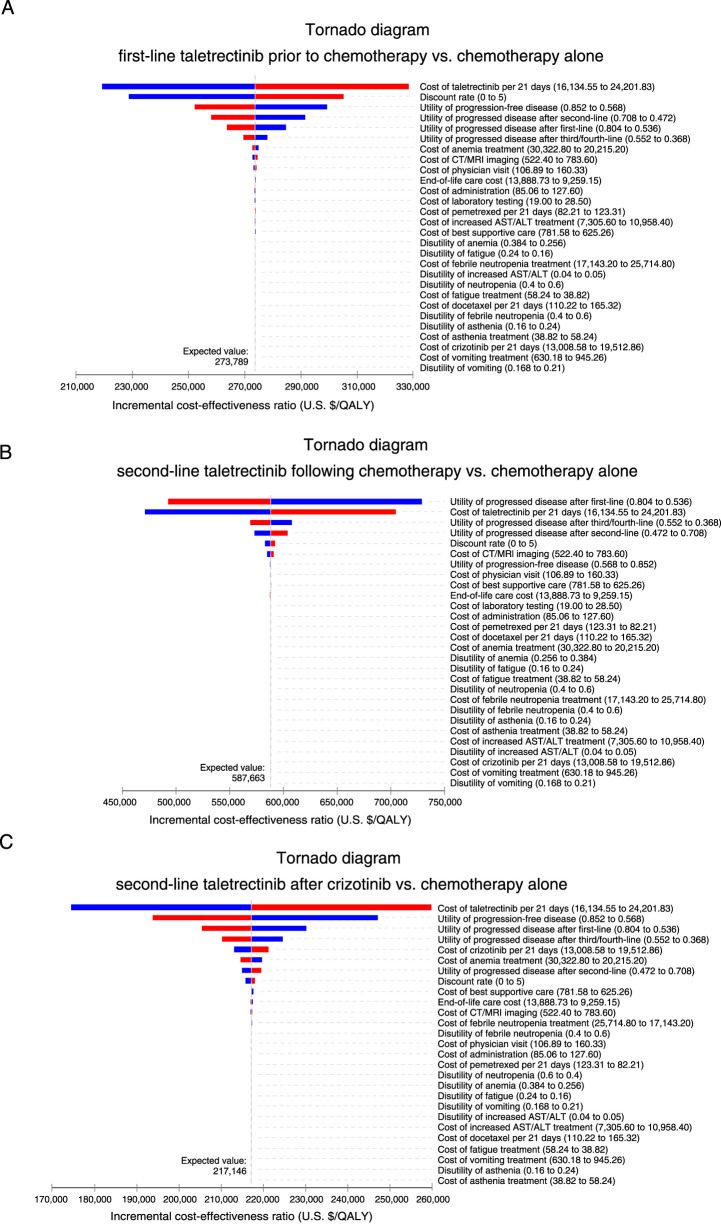
Univariate sensitivity analyses of the incremental cost-effectiveness ratio (ICER) are presented as tornado diagrams for: **(A)** first-line taletrectinib prior to chemotherapy vs. chemotherapy alone; **(B)** second-line taletrectinib following chemotherapy vs. chemotherapy alone; **(C)** second-line taletrectinib after crizotinib vs. chemotherapy alone.

Tornado plots illustrating the sensitivity of the ICERs to various parameters revealed that the cost of taletrectinib was consistently a key driver across all three taletrectinib-containing treatment regimens. More specifically: in the “first-line taletrectinib prior to chemotherapy” strategy (Figure 2A), the cost of taletrectinib and discount rate were the main influential factors. For “second-line taletrectinib following chemotherapy” (Figure 2B), the utility of progressed disease after first-line and the cost of taletrectinib were identified as the primary drivers. In the “second-line taletrectinib after crizotinib” strategy (Figure 2C), the cost of taletrectinib and the utility of progression-free disease were the most influential parameters.

Even when the values of these parameters were varied by ±20%, the ICER consistently remained above the $150,000 per QALY threshold, suggesting the robustness of the model’s conclusion.

**FIGURE 3:**
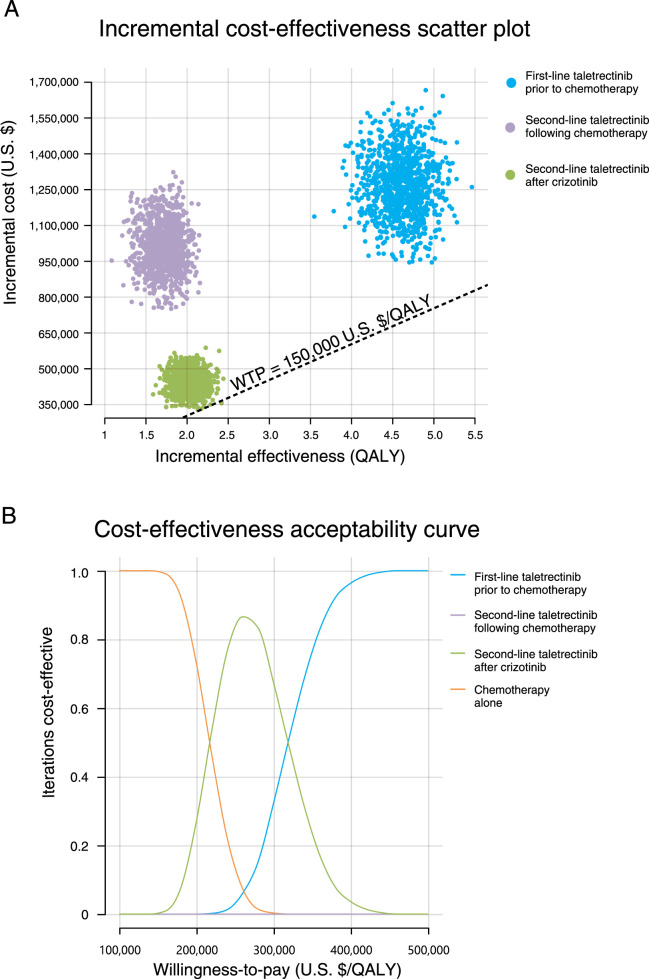
Sensitivity analyses: **(A)** incremental cost-effectiveness scatter plot for taletrectinib; **(B)** cost-effectiveness acceptability curves from probabilistic sensitivity analyses.

To conduct the probabilistic sensitivity analysis, 1,000 Monte Carlo simulations were performed. The resulting scatter plot, presented in Figure 3, showed that all simulated ICER estimates were located above the $150,000 per QALY WTP threshold. This finding suggests that while the three taletrectinib-containing treatment strategies provided improved therapeutic benefits compared with chemotherapy, they were also associated with a higher economic burden (Figure 3A).

At a WTP threshold of $150,000 per QALY, none of the three taletrectinib-containing treatment strategies were deemed cost-effective. However, as the WTP threshold increased, the most acceptable strategy shifted. Chemotherapy alone was first replaced by “second-line taletrectinib after crizotinib”, which was then superseded by “first-line taletrectinib prior to chemotherapy” with further increases in WTP (Figure 3B).

### Scenario analysis

**FIGURE 4:**
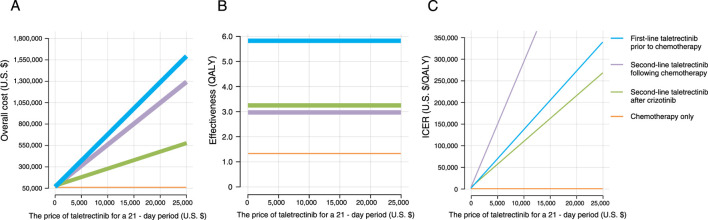
Overall cost **(A)**, effectiveness **(B)**, and incremental cost-effectiveness ratio (ICER) **(C)** of four treatment strategies across different taletrectinib prices.

Tornado plots revealed that the cost of taletrectinib was a key influential factor across all three taletrectinib-containing treatment regimens. To assess the sensitivity of the cost-effectiveness to taletrectinib cost, we analyzed the impact of varying the drug’s cost on the four treatment strategies’ cost-effectiveness. The base-case cost of taletrectinib was $20,168.19 per 21-day period; this cost was then varied from 0% to 120% of the base case value. The corresponding treatment costs for each strategy were then evaluated. As expected, the treatment costs for the three taletrectinib-containing strategies decreased as the price of taletrectinib was reduced. Figure 4A illustrates that the “first-line taletrectinib prior to chemotherapy” strategy consistently exhibited the highest treatment cost, while the “second-line taletrectinib after crizotinib” strategy consistently demonstrated the lowest.

Without considering economic factors, the clinical effectiveness of all four strategies remained unaffected by fluctuations in taletrectinib price (Figure 4B). The “first-line taletrectinib prior to chemotherapy” consistently showed the highest effectiveness. An ICER analysis was subsequently performed, comparing each strategy to chemotherapy alone, to identify the most economically favorable strategy across various taletrectinib prices. The analysis indicated that the “second-line taletrectinib after crizotinib” strategy was the most cost-effective, regardless of price changes, while the “second-line taletrectinib following chemotherapy” was always the least cost-effective (Figure 4C).

Cost-effectiveness was further assessed using a WTP threshold of $150,000. Under this framework, the maximum acceptable prices of taletrectinib were determined: for the “second-line taletrectinib after crizotinib” regimen, no more than $13,812.12 per 21-day period; for the “first-line taletrectinib prior to chemotherapy”, no more than $11,010.17 per 21-day period; and for the “second-line taletrectinib following chemotherapy”, under $5,047.54 per 21-day period (Figure 4C).

## Discussion

Given the advancements in targeted therapies, addressing the treatment needs of patients with ROS1+ NSCLC has become increasingly important. However, its rarity (1%–2% of NSCLC cases), combined with the high cost of ROS1-targeted drugs within the U.S. healthcare system, presents significant challenges to patient access and affordability (Sirrs et al., 2023). These high costs often hinder over half of patients with relevant genetic mutations from receiving recommended targeted therapies, sometimes forcing reliance on chemotherapy (Singal et al., 2019). Moreover, targeted therapies themselves can be complex, demanding frequent dose adjustments and treatment interruptions (Drilon et al., 2018).

To our knowledge, no published cost-effectiveness analysis of taletrectinib has been conducted. Prior pharmacoeconomic studies of other ROS1-targeted therapies have raised concerns: crizotinib was not cost-effective at 2021 Canadian prices (Beca et al., 2021), while entrectinib and repotrectinib similarly failed to demonstrate cost-effectiveness as either first- or second-line treatments in the U.S. (Huo et al., 2024; Huo and Chen, 2026). However, given the distinct efficacy profile and prolonged progression-free survival observed with taletrectinib, its economic value remains uncertain. Therefore, a comprehensive cost-effectiveness evaluation is warranted to inform value-based reimbursement and coverage decisions within the U.S. healthcare system.

Our analysis revealed that despite taletrectinib’s clinical benefits in both first-line and second-line settings, are associated with a substantially higher cost per QALY. Within the context of the U.S. healthcare system, oncology drugs are typically reimbursed through a multi-payer structure involving private insurers, Medicare, and Medicaid. For most targeted oral anticancer therapies, coverage is provided through pharmacy benefit programs such as Medicare Part D or commercial insurance formularies, where high drug prices may translate into substantial expenditures for both payers and patients through coinsurance and cost-sharing mechanisms. In this environment, payers increasingly rely on value-based assessments to inform coverage decisions. Although the United States does not have a formal national health technology assessment authority, WTP thresholds of $100,000–$150,000 per QALY are widely used in economic evaluations and by organizations such as the Institute for Clinical and Economic Review to assess the value of new therapies. At the commonly cited upper threshold of $150,000 per QALY, our analysis indicates that taletrectinib is not cost-effective across any of the evaluated strategies, including first-line use and second-line therapy following crizotinib or chemotherapy. These findings suggest that under current U.S. drug pricing and reimbursement conditions, the additional health benefits associated with taletrectinib may not justify its high treatment costs from a payer perspective. In practice, treatments that exceed commonly accepted cost-effectiveness thresholds may face restricted coverage, prior authorization requirements, or step-therapy policies, potentially limiting patient access despite demonstrated clinical efficacy.

Taletrectinib price was the principal driver of cost-effectiveness in our model. Scenario analysis demonstrated that lower drug prices were consistently associated with lower ICERs, underscoring the high price sensitivity of taletrectinib-based strategies in the U.S. reimbursement environment. This issue is especially relevant for the first-line taletrectinib followed by chemotherapy regimen, which produced the greatest clinical benefit among all taletrectinib-containing strategies evaluated. Nevertheless, in the United States, actual affordability and uptake are shaped not only by clinical value but also by payer reimbursement, formulary positioning, prior authorization, and patient cost-sharing obligations. Using a WTP threshold of $150,000 per QALY, our analysis indicates that this regimen would be cost-effective only if the maximum reimbursable price of taletrectinib were no greater than $11,010.17 per 21-day cycle. Accordingly, despite its superior effectiveness, this strategy is unlikely to meet conventional U.S. cost-effectiveness criteria unless meaningful reductions in the effective reimbursed price can be achieved.

Despite these economic findings, taletrectinib represents an effective treatment option for ROS1-positive NSCLC patients, supported by compelling clinical efficacy demonstrated in the TRUST trial and its inclusion in NCCN guideline recommendations. In the United States, however, the translation of clinical benefit into real-world patient access is strongly influenced by the structure of the drug pricing and reimbursement system. Oncology drugs such as taletrectinib are typically reimbursed through a combination of private commercial insurance, Medicare Part D or Medicare Advantage prescription drug plans, and Medicaid. Coverage decisions often depend on formulary placement, prior authorization requirements, and payer assessments of clinical value and budget impact. Although guideline inclusion generally facilitates insurance coverage, many targeted oncology therapies are placed on specialty tiers within pharmacy benefit plans, which often require substantial patient cost sharing in the form of coinsurance rather than fixed copayments. Under Medicare Part D, for example, patients may face significant out-of-pocket costs during the deductible and initial coverage phases before reaching catastrophic coverage thresholds. Similarly, private insurers frequently require prior authorization and may implement step therapy policies to manage utilization and spending on high-cost targeted agents. As a result, even when a therapy is clinically recommended and formally covered, patients may still experience financial barriers that limit timely access. Against this reimbursement backdrop, the high acquisition cost of taletrectinib raises concerns regarding its affordability and overall economic value. High drug prices can impose substantial financial pressure on both patients and healthcare payers, including private insurers, Medicare Part D plans, and Medicaid programs. As precision oncology continues to expand, cumulative spending on targeted therapies may further increase overall oncology-related healthcare expenditures in the United States. Consequently, despite its meaningful clinical benefits, the high treatment cost of taletrectinib may limit its accessibility and cost-effectiveness in routine practice, underscoring the importance of pricing strategies, payer negotiations, and patient assistance programs to improve equitable patient access.

Given the current price of taletrectinib and the low likelihood of immediate price reductions, achieving both high efficacy and acceptable cost-effectiveness simultaneously appears challenging. This necessitates a personalized treatment approach that prioritizes individual patient needs and circumstances. Based on our results, patients prioritizing maximum QALY gains and possessing sufficient financial resources may prefer first-line taletrectinib followed by chemotherapy. Conversely, for patients facing financial constraints or within healthcare systems emphasizing cost-effectiveness, second-line taletrectinib following crizotinib may represent a more pragmatic and affordable strategy.

The significant research and development investments required for targeted cancer therapies contribute to their high prices. This creates a societal dilemma: while lower prices could impede cost recovery, the escalating costs of new cancer drugs pose a major challenge to healthcare systems, particularly in countries with limited cost controls (Giuliani and Bonetti, 2016). Addressing this necessitates greater transparency and fairer pricing within the pharmaceutical industry (Annett, 2021). Drug companies should disclose research and development spending and establish prices that reflect both clinical benefits and overall societal costs, ultimately pursuing fair, value-based pricing models (Giuliani and Bonetti, 2016; Bonetti and Giuliani, 2021).

This study has several limitations, partly attributable to data availability and key model assumptions (Husereau et al., 2013). First, indirect costs, such as productivity losses, were not included in the analysis. However, direct medical costs are typically prioritized by hospital decision-makers and clinicians, as these expenses directly influence treatment choices and healthcare budget management. Second, treatment options and outcomes may vary considerably across individual patient profiles, highlighting the need for real-world evidence to further validate long-term model outcomes. Third, the costs associated with mild (grade 1 or 2) adverse events were not included in the analysis. Although this may have slightly overestimated the cost-effectiveness of taletrectinib, one-way sensitivity analyses indicated that these costs were minimal and therefore unlikely to substantially affect the overall results. Fourth, the treatment sequence in which taletrectinib is used as first-line therapy followed by crizotinib was not evaluated, as no clinical trial data with survival outcomes are currently available for crizotinib used as second-line therapy in this specific sequence. Future studies incorporating real-world data may help to further evaluate this treatment strategy. Additionally, although taletrectinib was approved in China in December 2024, the present analysis was conducted from the perspective of the U.S. healthcare system rather than the Chinese setting. Currently, the market price of taletrectinib in China is approximately RMB 48,600 per month (about USD 7,086.41), which remains beyond the affordability range of many Chinese households, and its reimbursement status within the national medical insurance system is still evolving. Furthermore, currency conversion between Chinese yuan and U.S. dollars may introduce additional uncertainty due to exchange rate fluctuations and differences in healthcare cost structures between countries. Importantly, the findings of this study should therefore be interpreted within the context of the U.S. healthcare system, including U.S. drug pricing structures and commonly applied willingness-to-pay thresholds. Consequently, the cost-effectiveness results and policy implications may not be directly generalizable to countries with different healthcare systems, drug pricing regulations, or reimbursement mechanisms. Future research should evaluate the economic value of taletrectinib in other healthcare settings where drug prices, reimbursement policies, and WTP thresholds may differ substantially from those in the U.S.

## Conclusion

Taletrectinib has demonstrated substantial efficacy in the treatment of ROS1-positive NSCLC. However, based on current pricing, none of the taletrectinib-containing strategies, whether used as first-line therapy or as second-line treatment following crizotinib or chemotherapy, were cost-effective for patients with ROS1+ NSCLC compared to chemotherapy alone, as their ICERs exceeded the established WTP threshold, indicating higher costs per QALY from the perspective of U.S. healthcare payers. Nevertheless, considering individual patient priorities, a personalized approach to treatment decisions can be adopted. For patients who prioritize maximizing QALYs and have sufficient financial resources, first-line taletrectinib may be a preferred option. Conversely, for those with limited financial capacity or in contexts prioritizing cost-containment, second-line taletrectinib after crizotinib could be more suitable.

## Data Availability

The original contributions presented in the study are included in the article/Supplementary Material, further inquiries can be directed to the corresponding authors.
