# Supplementary material for: Taletrectinib in ROS1+ non–small cell lung cancer: a cost-effectiveness analysis in the United States
Source: Front Pharmacol. 2026 Apr 13;17:1684603. doi: 10.3389/fphar.2026.1684603 (PMC13111073; doi:10.3389/fphar.2026.1684603)
Supplement: Supplementary file 1 [file Table1.doc]

Table S1 AIC/BIC values for different distributions in various trials.

| Distribution | Taletrectinib  1st-line | | Taletrectinib  2nd-line | | Crizotinib | | Pemetrexed | | Docetaxel | | BSC | |
| --- | --- | --- | --- | --- | --- | --- | --- | --- | --- | --- | --- | --- |
|  | AIC | BIC | AIC | BIC | AIC | BIC | AIC | BIC | AIC | BIC | AIC | BIC |
| Exponential | 558.7 | 561.7 | 522.6 | 525.3 | 334.7 | 336.6 | 1,291.2 | 1,295.0 | 278.9 | 280.9 | 247.3 | 249.2 |
| Gamma | 560.7 | 566.8 | 524.0 | 529.5 | 335.9 | 339.8 | 1,117.4 | 1,125.1 | 274.5 | 278.5 | 241.2 | 245.0 |
| Gen. gamma | 561.6 | 570.8 | 514.7 | 522.9 | 332.0 | 337.9 | 1,073.0 | 1,084.6 | 276.5 | 282.5 | 236.9 | 242.5 |
| Gompertz | 560.7 | 566.8 | 523.5 | 528.9 | 331.7 | 335.6 | 1,213.9 | 1,221.7 | 279.0 | 283.1 | 249.3 | 253.0 |
| Weibull | 560.7 | 566.8 | 524.5 | 530.0 | 335.2 | 339.1 | 1,150.0 | 1,157.8 | 275.2 | 279.2 | 244.6 | 248.4 |
| Log-logistic | 561.2 | 567.4 | 518.0 | 523.5 | 331.6 | 335.5 | 1,104.4 | 1,112.2 | 278.3 | 282.3 | 232.5 | 236.3 |
| Log-normal | 559.7 | 565.8 | 514.4 | 519.8 | 330.5 | 334.5 | 1,092.5 | 1,100.3 | 281.1 | 285.1 | 235.1 | 238.9 |

AIC, Akaike information criteria; BIC, Bayesian information criteria; BSC, best supportive care.

**Table S2** Background mortality rate.

| Age | Background mortality rate | Age | Background mortality rate | Age | Background mortality rate |
| --- | --- | --- | --- | --- | --- |
| 50 | 0.004515 | 67 | 0.016329 | 84 | 0.076869 |
| 51 | 0.004833 | 68 | 0.017524 | 85 | 0.086054 |
| 52 | 0.005204 | 69 | 0.018824 | 86 | 0.094545 |
| 53 | 0.005647 | 70 | 0.020179 | 87 | 0.106195 |
| 54 | 0.006162 | 71 | 0.021711 | 88 | 0.118983 |
| 55 | 0.006709 | 72 | 0.023521 | 89 | 0.132946 |
| 56 | 0.007285 | 73 | 0.025618 | 90 | 0.148104 |
| 57 | 0.007930 | 74 | 0.028122 | 91 | 0.164457 |
| 58 | 0.008641 | 75 | 0.030467 | 92 | 0.181983 |
| 59 | 0.009392 | 76 | 0.034339 | 93 | 0.200630 |
| 60 | 0.010173 | 77 | 0.037380 | 94 | 0.220320 |
| 61 | 0.010960 | 78 | 0.041500 | 95 | 0.240942 |
| 62 | 0.011741 | 79 | 0.045247 | 96 | 0.262361 |
| 63 | 0.012524 | 80 | 0.050833 | 97 | 0.284411 |
| 64 | 0.013340 | 81 | 0.056254 | 98 | 0.306908 |
| 65 | 0.014218 | 82 | 0.062471 | 99 | 0.329650 |
| 66 | 0.015280 | 83 | 0.069526 | 100 | 1.000000 |

Table S3 Discrepancies between clinical trials and the estimated model.

| Treatment | Model (months) | Clinical trials (months) |
| --- | --- | --- |
| Taletrectinib 1st-line | 45.65 | 45.6 |
| Taletrectinib 2nd-line | 9.68 | 9.7 |
| Crizotinib | 19.39 | 19.3 |
| Pemetrexed | 6.92 | 6.9 |
| Docetaxel | 3.90 | 3.9 |
| BSC | 4.63 | 4.6 |

BSC, best supportive care.

Table S4 Formulas used in Monte Carlo simulations for different distributions.

| Distribution | Formula |
| --- | --- |
| Gamma | 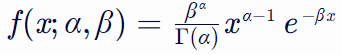, 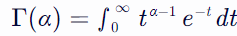 |
| Beta | 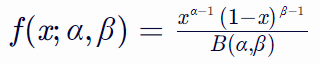, 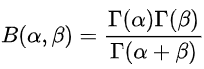, 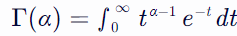 |
| Normal | 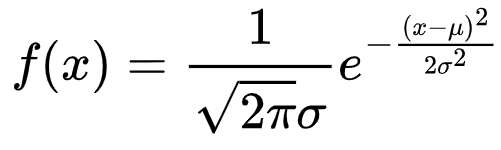 |
