# Supplementary material for: Taletrectinib in ROS1+ non–small cell lung cancer: a cost-effectiveness analysis in the United States
Source: Front Pharmacol. 2026 Apr 13;17:1684603. doi: 10.3389/fphar.2026.1684603 (PMC13111073; doi:10.3389/fphar.2026.1684603)
Supplement: Supplementary file 2 [file DataSheet1.pdf]

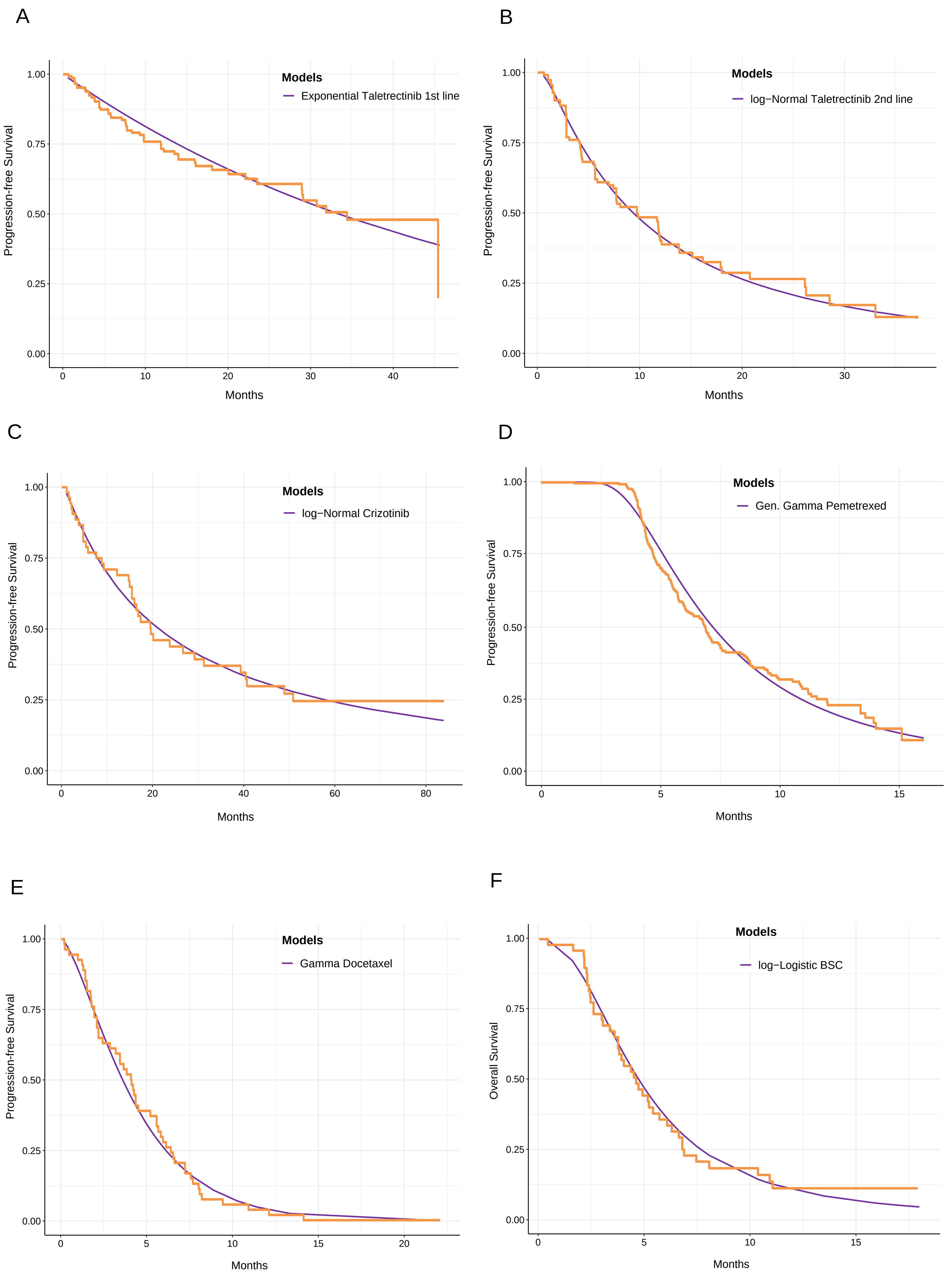

Figure S1. Fitting of parametric distributions in various trials. (A) Taletrectinib, 1st line; (B) Taletrectinib, 2nd line; (C) Crizotinib; (D) Pemetrexed; (E) Docetaxel; (F) Best supportive care.
